# Preparation, characterization, and life cycle assessment of banana rachis-recycled high-density polyethylene composites

**DOI:** 10.1038/s41598-023-42613-0

**Published:** 2023-10-02

**Authors:** Demis Cabrera, Haci Baykara, Ariel Riofrio, Mauricio Cornejo, Julio Cáceres

**Affiliations:** 1 Faculty of Mechanical Engineering and Production Science (FIMCP), ESPOL Polytechnic University, P.O. Box 09-01-5863, Guayaquil, Ecuador; 2 Plastics Processing Laboratory (PPL), ESPOL Polytechnic University, P.O. Box 09-01-5863, Guayaquil, Ecuador; 3 Nanotechnology Research and Development Center (CIDNA), ESPOL Polytechnic University, P.O. Box 09-01-5863, Guayaquil, Ecuador

**Keywords:** Characterization and analytical techniques, Chemical engineering, Mechanical properties, Environmental monitoring

## Abstract

Agro-industrial wastes are sustainable resources that have advantages as a reinforcement for polymeric matrices. This study examined the use of banana rachis fiber (BRF) in reinforcing the recycled high-density polyethylene (rHDPE) matrix. For this purpose, polymer composites with 5–20 wt% of BRF were prepared by the extrusion process using a twin-screw extruder and followed a hot press method. The structure of rHDPE/BRF composites and their characteristic peaks of degradation were successfully identified by the Fourier-transformed infrared spectroscopy and thermogravimetric analysis techniques, respectively, revealing a good dispersion of BRF in rHDPE. Differential scanning calorimetry results of the composites demonstrated that melt enthalpy decreases as the amount of BRF increases. XRD diffractograms revealed a crystallinity reduction of rHDPE due to the increase of fiber within the polymer matrix, which is reflected in the characteristic peaks' intensity decrease of HDPE. Variation in thermal and chemical properties with the addition of BRF in rHDPE was successfully evaluated in this study. Life cycle assessment for 1 kg composite production has also been evaluated. The banana rachis-rHDPE composite materials reduce the overall environmental impacts when the filler concentration increases.

## Introduction

In the composite manufacturing process, natural fibers are a potential and renewable resource that offers various advantages as reinforcement of polymeric matrices^1^ such as high rigidity, lightweight, high durability, and water resistance^2–6^. There are various published papers in the literature related to natural fibers-reinforced polymer composites^1,7–11^. The use of natural fibers has been favorable in terms of mechanical, morphological, and permeability properties and an economical raw material to be acquired from agro-industrial waste.

Ecuador is an agro-industrial country. After shrimp, bananas are the second product to be exported nationally^12^ a large amount of organic waste, such as banana rachis. This waste has been used in some industries as biomass^13^. However, the waste management hierarchy, an internationally accepted guide that ranks waste management practices in ascending order from most to least preferred, maintains that agro-industrial waste is better used as raw material and biomass^14^. So, finding an application that takes advantage of this waste and does not generate environmental impact like biomass is necessary and crucial.

The use of plastics has generated a large amount of waste every day. It represents a vast majority of the solid waste found in landfills causing significant pollution at a global scale^15^. Ecuador only recycles 4% of all solid waste generated^16^. On the other hand, United Nations revealed that only 9% of all plastic waste is recycled worldwide. The remaining residue gets burned and expelled into the environment generating greenhouse gases (GHG) and polluting the planet's flora and fauna^17,18^. The selection of recycled polymers as raw materials encourages the new circular economy trend by reincorporating non-organic waste, such as plastic, into new industrial processes to create new materials for new products.

There are some studies^19,20^ that reported the application of banana fibers in the reinforcement of polymers, but none specifically with BRF. Neher et al.^19^ evaluated the physical, mechanical, and thermal properties of rHDPE composites reinforced with banana stem fibers. The study revealed greater mechanical strength and thermal stability in composites with a higher percentage of fiber. However, the technique of direct die-casting was used for the different test specimen formations without prior extruder processing. This process is essential since it ensures the appropriate fiber dispersion within the polymer matrix and is a method that the polymer/plastic industry always carries out. Ogunsile et al*.*^20^ assessed the use of banana stalk fiber in low-density polyethylene from 5 to 20 vol%. Results showed a good interfacial bonding between the matrix and filler, with an improvement in tensile strength.

This study aims to prepare and characterize recycled rHDPE reinforced with 5, 10, 15, and 20 wt% BRF obtained from the coastal region of Ecuador. Tensile strength, elongation, stiffness, impact stress, thermal properties such as melting and crystallization temperature, and rheological and morphological properties of rHDPE-banana rachis composites have been evaluated. The raw material, banana rachis, and the corresponding composites have been characterized using FTIR, TGA–DSC, SEM, and rheometry techniques. The environmental impact of rHDPE-banana rachis composites has been investigated using life cycle assessment.

## Materials and methods

### Materials

rHDPE-banana rachis composites have been prepared using BRF obtained from the agroindustry in Guayas-Ecuador and recycled-high density polyethylene. The rHDPE was supplied from the recycled industry "NUTEC", which reprocessed the drawers of water bottles to obtain the recycled polymer.

### Fiber preparation

The BRFs were received in long strips, dried for 24 h at room temperature, then dried and milled in a pelletizer to obtain short fibers of approximately 5 mm. Grounded fibers were dried again in a 220 V SECAGEM Q317M-33 model oven at 60 °C for 24 h, as reported in the literature^1,19^. The dried grounded fibers were pulverized in a ball mill at 450 rpm for 5 min to reduce the particle size to 240 μm.

### rHDPE-BRF composite preparation

The mixing process between rHDPE and BRF was carried out in the torque rheometer Brabender Plastograph EC Plus. Before mixing the fiber with the rHDPE, it was dried again for 24 h due to the high hydrophilicity of BRF^2–4^. Five different composites were prepared using 0, 5, 10, 15, and 20 wt% of dried BRF concerning the weight of rHDPE. The formulations of each composite can be seen in Table 1. As the maximum capacity of the equipment was 35 g, each formulation was prepared considering the capacity of the equipment. The raw materials were put in the hopper of the Brabender and mixed at 170 °C with a screw speed of 60 rpm for 15 min^1,2^.

**Table 1:** Mix proportions for BRF and rHDPE composites.

Sample	Formulations	
	rHDPE, g (%)	BRF, g (%)
rHDPE 100	35 (100)	–
rHDPE + BRF 95/5	33.25 (95)	1.75 (5)
rHDPE + BRF 90/10	31.5 (90)	3.5 (10)
rHDPE + BRF ​85/15	29.75 (85)	5.25 (15)
rHDPE + BRF 80/20	28 (80)	7 (20)

## Mechanical properties

### Mechanical test specimen preparation

The extruded product was manually cut into small pellets and put into molds for tensile and impact specimen preparation by hot compression using a thermal plate press with a temperature and pressure of 170 °C and 220 Pa, respectively, for 10 min. Before that, the pellets were dried again due to the hydrophilicity of BRF. After all hot compression, the specimens were cooled (still in compression) until a temperature of 110 °C. Subsequently, they were rapidly cooled down to room temperature by immersing them in water.

### Mechanical test

Ten specimens for each mechanical test (tensile and impact) were made for each formulation. For the tensile test, the universal testing machine was used under the ASTM D638^21^ standard at a speed of 500 mm min^−1^, where Young's modulus, tensile strength, elongation, and fracture strength were determined. Impact tests were carried out following the ASTM D256^22^ standard norm.

### Differential scanning calorimetry (DSC)

Melting and recrystallization temperatures were analyzed using a TA Instruments DSC, model Q200. Tzero aluminum hermetic pans were used for both the samples and the reference. The samples were analyzed between 23 and 200 °C at 10 °C min^−1^ of the heating ramp and under the nitrogen atmosphere of 50 mL min^−1^. A double-sweep test was carried out for each sample to obtain more accurate results. Crystallinity's percentage (%${X}_{c}$) was calculated using the following equation (Eq. 1) given in literature^23^:

1 $${X}_{c}=\left(\frac{\Delta {H}_{m}}{\Delta {H}_{mt}\times w\%}\right)\times 100$$

where $\Delta {H}_{m}$ is the melting enthalpy of rHDPE in the composite determined from the DSC test, $\Delta {H}_{mt}$ is the melting enthalpy of pure PE (100% crystalline), i.e., 295.8 J g^−1^^24^, and w% is the weight percentage of PE in the composite^25,26^. The following equation (Eq. 2) is another alternative reported in the literature to calculate the degree of crystallinity^27,28^.

2 $$\%{X}_{c}=\left(\frac{\Delta {H}_{m}-\Delta {H}_{c}}{\Delta {H}_{mt} \times w\%}\right)\times 100$$

where $\Delta {H}_{c}$ is the crystallization enthalpy of rHDPE in the composite determined from the DSC test.

### Fourier transform infrared spectroscopy (FTIR) analysis

The functional groups in the different samples were identified using a Perkin Elmer Spectrum 100 spectrophotometer. For this test, films were prepared for each composite sample by hot pressing 1 g of the sample at a temperature and pressure of 170 °C and 220 Pa, respectively, for 3 min.

### X-ray diffraction (XRD)

X-ray diffraction properties were tested using a Phillips PANanalytical X'pert-PRO model X-ray diffractometer with CuKα radiation at 40 kV–30 mA. The samples for the respective tests were taken from the discarded mechanical specimens.

### Thermogravimetric analysis (TGA)

A TA Instruments Q600 SDT carried out the characterization for each sample under an ultrapure nitrogen medium. For this test, 5–15 mg of each sample were settled in an alumina crucible and then exposed from room temperature to 1000 °C at a heating ramp of 10 °C min^−1^.

### Rheological analysis

Material fluidity was analyzed using a Malvern Kinexus Pro + Rotational Rheometer with two parallel plates with a 1.85 mm gap setting at 180 °C and a shear rate range between 0.1 and 100 s^−1^. Test specimens were hot compressed using 2 cm diameter disc molds.

### Scanning electron microscopy (SEM)

An FEI Inspect S50 with a BSED (Back Scattering Electron Detector) detector scanning electron microscope was used for the microstructural characterization of the composites. SEM analysis of each sample was done using a voltage of 10 kV and 3.5 Spot in low vacuum mode at a pressure of 12–15 Pa. For this purpose, each specimen's IZOD impact test cross-section was analyzed to observe the dispersion and morphology of these composite materials. The samples were coated with gold–palladium using an EMITECH SC7620 model sputter coater, applying 5 mbar of pressure and a current of 18 mA for 60 s.

### Life cycle assessment

**Figure 1:**
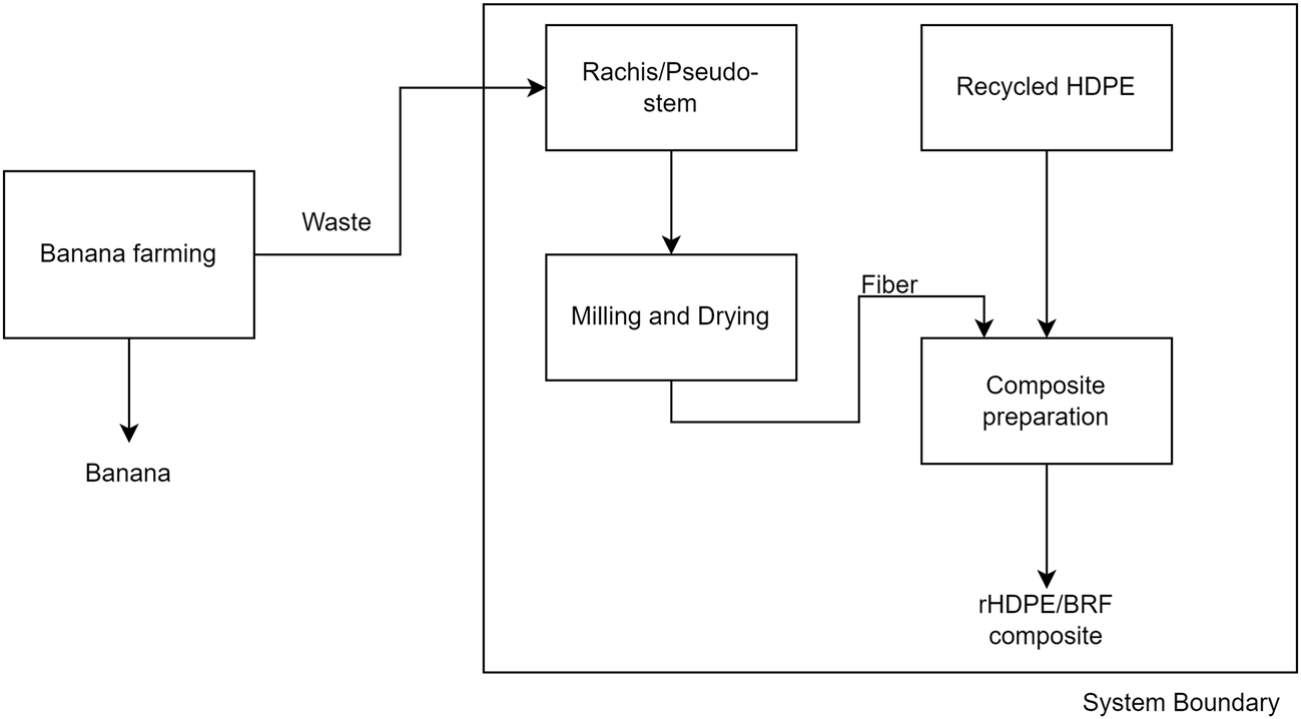
System boundaries considered for the inventory in the LCA.

The life cycle assessment is based on the ISO 14040 and 14044 norms^29,30^. It establishes the environmental impact of a product from a circular economy point of view. In this sense, the study aims to evaluate the environmental profile of the composite using banana rachis from Ecuador as a reinforcement of the polymer's mechanical properties. The LCA uses a cradle-to-gate perspective. The assessment covers the impacts of composite production using recycled HDPE (rHDPE) and banana rachis as a by-product of the banana industry. The scope of the analysis involves five different scenarios: 0, 5, 10, 15, and 20% of banana rachis in the polymer matrix. Environmental indicators have been evaluated using the data given in Table 1 to obtain the results for comparison. The environmental study defines the functional unit as 1 kg of polymeric composite production. Figure 1 shows the system boundary. The processes included in the assessment are depicted within the box that represents the limits of the modeling.

For the inventory analysis and later conversion to environmental impact indicators, SimaPro software^31^ was used. The method used for estimations is ReCiPe Midpoint H^32^. The method provides data from across 18 different environmental categories. This allows for a better understanding of the environmental profile of the composite.

A library for the electricity of Ecuador was included in the software^33^. The inventory included a medium voltage supply and considered a low voltage conversion in the factory of the composite. In addition, the following library from the Ecoinvent 3.7^34^ database was used: Polyethylene, high density, granulate, recycled {Rest of the World}|Cut-off, U. Also, from the Agri Footprint 5.0, the transport was estimated using the inventory Transport, truck < 10 t, EURO3, 50%LF, empty return/GLO Mass.

The banana farms are located in three different provinces Guayas (76 km), Los Rios (177 km), and El Oro (196 km). The banana rachis or pseudostem used in this study was obtained from a farm in Guayas. However, the distance was calculated as the average from the farms, an estimated distance of 149.7 km.

Also, the rachis is assumed not to add pollution to the environment because the main product of banana farming is the fruit. The stem is cut down and used as a natural fertilizer for the soil. A better application is evaluated for this waste. The use of energy for fiber treatment (drying and milling) and the energy used for the composites were considered as mentioned in the "Fiber preparation" and "rHDPE/BRF composite process" subsections. In addition, the carbon dioxide sequestrated from the fibers has been considered in the data. According to a study investigating the carbon abatement potential of banana crops in Ecuador, the banana rachis retains about 0.4 kg of CO_2_ per kg of the banana rachis.

## Results and discussion

### Differential scanning calorimetry (DSC)

**Figure 2:**
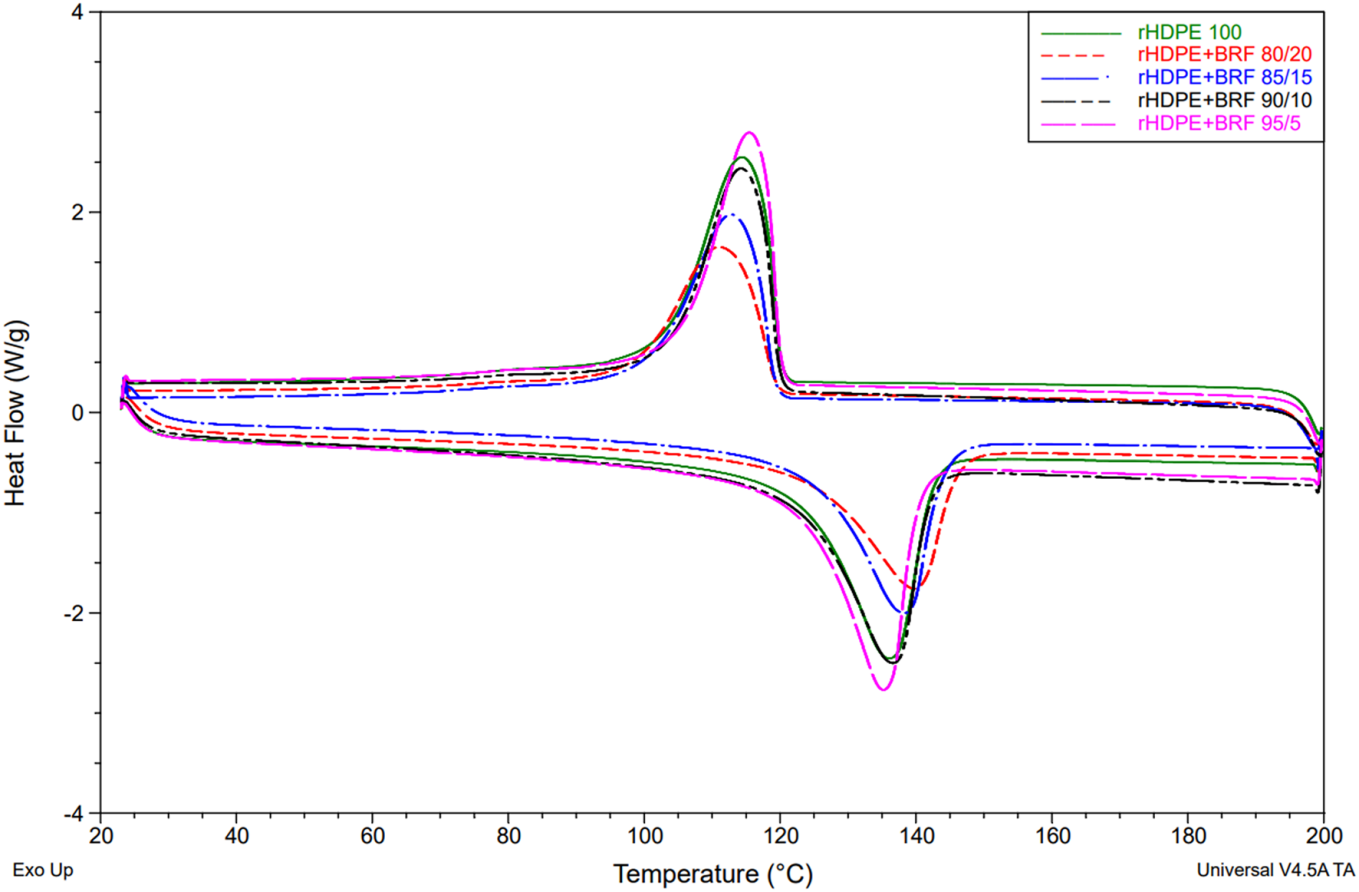
Comparison of all the DSC results obtained for the different formulations.

Table 2 and Fig. 2 present the DSC results. Table 2 shows that the incorporation of BRF slightly increases the melting temperature of the composite but decreases its crystallization temperature.

**Table 2:** DSC results for rHDPE and corresponding composites.

Sample	Melting temperature (°C)	Melting enthalpy (J g^−1^)	Crystallization temperature (°C)	Crystallization enthalpy (J g^−1^)	Crystallinity percentage (%)
rHDPE 100	136.07	168.50	114.59	149.80	56.96
rHDPE + BRF 95/5	135.15	159.30	115.53	149.50	56.69
rHDPE + BRF 90/10	136.51	147.90	114.33	141.00	55.56
rHDPE + BRF 85/15	139.16	141.00	112.86	130.30	56.08
rHDPE + BRF 80/20	139.68	123.00	111.47	114.00	51.98

Furthermore, as observed in Table 2, crystallization and melting enthalpies decrease, as well as the percentage of crystallinity, when the amount of BRF is increased, similar to a study^35^ related to banana stem fiber-reinforced rHDPE composites. HDPE is a moderately flexible polymer, whereas untreated BRF is a more rigid material. As BRF increased in the HDPE matrix, its mobility decreased and hindered the rearrangement of the rHDPE microstructure. Consequently, the rHDPE–BRF composites demanded less heat to melt^36^.

BFR is an amorphous material, so its increase within the polymeric matrix will decrease the crystallinity of the composite material, as seen in Table 2. rHDPE100 sample showed 56.96% crystallinity which is consistent with the value reported in the literature^35^. On the other hand, the rHDPE + BRF 80/20 sample containing 20% BRF presented 51.98% crystallinity. A graphic description of melting and crystallization enthalpies is shown in Fig. 2.

### FTIR analysis

**Figure 3:**
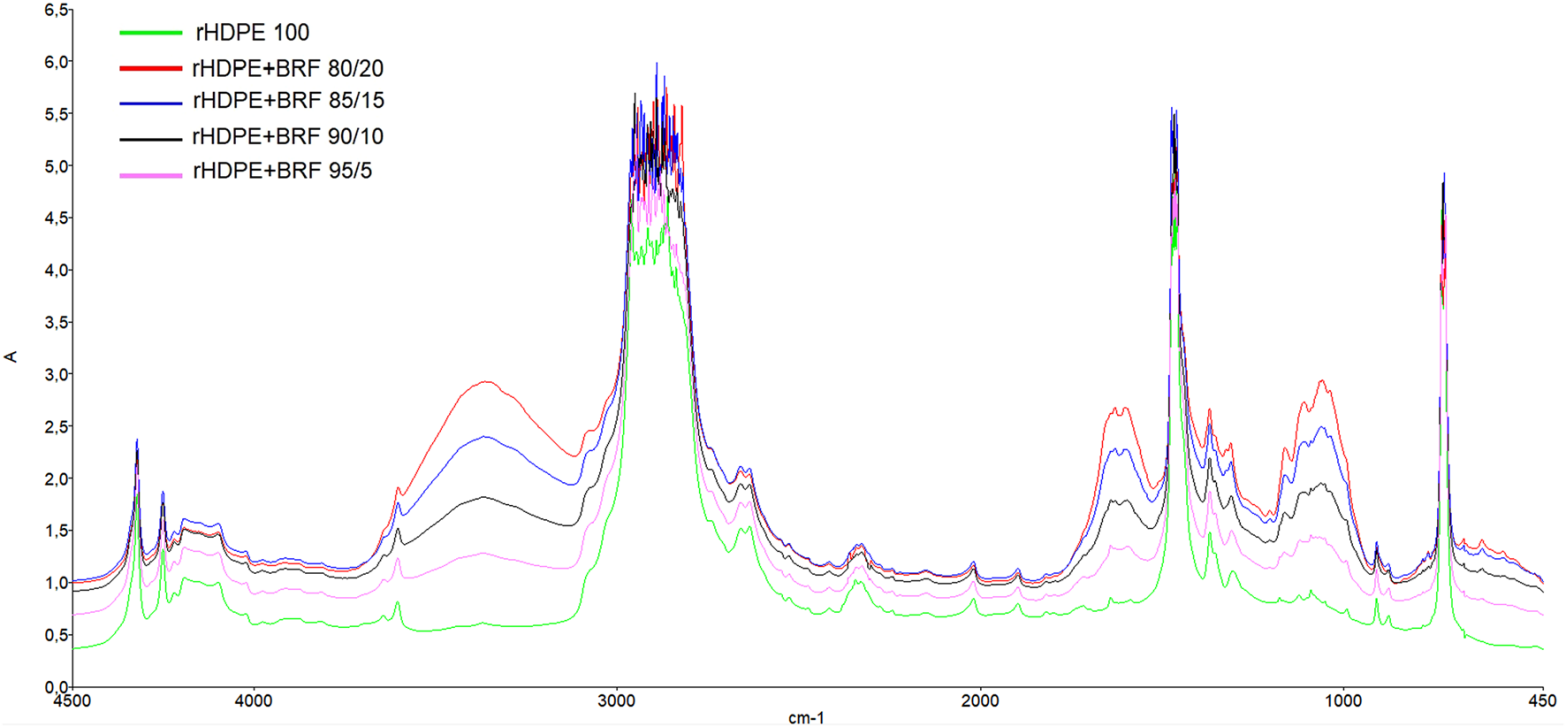
Comparison of all the FTIR results obtained for the different formulations.

Figure 3 shows the characteristic peaks of high-density polyethylene functional groups. As seen in the figure, a strong peak (1) between 3000 and 2800 cm^−1^ due to saturated C–H stretching, (2) the bands between 1472 and 1460 cm^−1^ for methylene group' scissoring and (3) the peaks between 732 and 720 cm^−1^ due methylene rocking which confirm the structure of the polyethylene have been observed^26,37,38^.

Figure S1 in Supplementary Information File shows the BRF characteristic peaks. These are (1) the medium broad band on 3500–3400 cm^−1^ due to O–H stretching vibration of the intermolecular hydrogen bonding associated with the main organic constituents of pseudo stems (cellulose, hemicellulose, and lignin)^20,39–42^, (2) the weak peak at 2923.73 cm^−1^ due to the asymmetric and symmetrical stretching modes of aliphatic C–H present in cellulose and hemicellulose^20,39,43^, (3) the strong peak at 1637.9 cm^−1^ due to the O–H bending vibrations of absorbed water^20,39,41,43^, (4) The absorption bands near 1415.32 cm^−1^ and 1384.65 cm^−1^ due to –CH_2_ symmetric bending in lignin, cellulose, and hemicellulose, and –CH symmetric deformation of cellulose and hemicellulose, respectively^20,39,43^, (5) the band at 1330.25 cm^−1^ associated with the –OH in-plane bending vibration in cellulose^39,40^, (6) the low-intensity peaks between 1109 and 1057.09 cm^−1^ due to the C–O–C pyranose ring skeletal vibration of cellulose and the asymmetric stretching of C–O–C in the cellulose and hemicelluloses^39,40^, (7) the signatures peak at 1162.27 and 904.46 cm^−1^ attributed to C–O–C symmetric stretching of β-glycosidic linkages of the cellulosic material^20,39,42,43^, (8) the band located in the range of 698.64–542.88 cm^−1^ due to C–OH out-of-plane deformation in cellulose^39^.

Figure 3 shows how the characteristic peaks of HDPE decrease in intensity as the addition of BRF increases. The absorption bands in the range of 3000–2800 cm^−1^ do not show an apparent decrease because the BRF also presents a characteristic peak in the same region. Therefore, an increase in the intensity of the bands is detected. Likewise, the characteristic peaks of the BRF (the peak around 3400, 1637.9 cm^−1^) are more visible as the amount of filler in the polymer matrix increases. These results are as expected and demonstrate the excellent dispersion of the fiber in HDPE. A decrease in the characteristic peaks of HDPE indicates a reduction in the material's crystallinity since the semi-crystalline base polymer gives the crystallinity. The increase of BRF in composites probably causes a decrease in crystallinity. The compare option of the FTIR software presented the similarity between the composites and pure polyethylene, as seen in Figure S2 in the Supplementary Information File. The similarity results of the compare option showed that the decrease in the percentage of rHDPE in composite decreases the similarity between the sample and pure HDPE existing in the software's database. This evidence supports what was mentioned above regarding crystallinity as the percentage of BRF increases, which is consistent with the results obtained by XRD (Table 4) and DSC (Table 2).

Additionally, the theoretical thicknesses of the BRF-rHDPE composites were calculated using implementing the equation given in the literature^44^ using FTIR spectra and a refraction index of 1.57^45,46^. The theoretical thicknesses of the samples were found to be around 0.07 mm. The experimental results of the thickness of the films gave an average of 0.3 mm. Experimental thicknesses of the films were measured using a REXBETI (0–25 mm, 0.001 mm) digital micrometer.

### X-ray diffraction (XRD)

**Figure 4:**
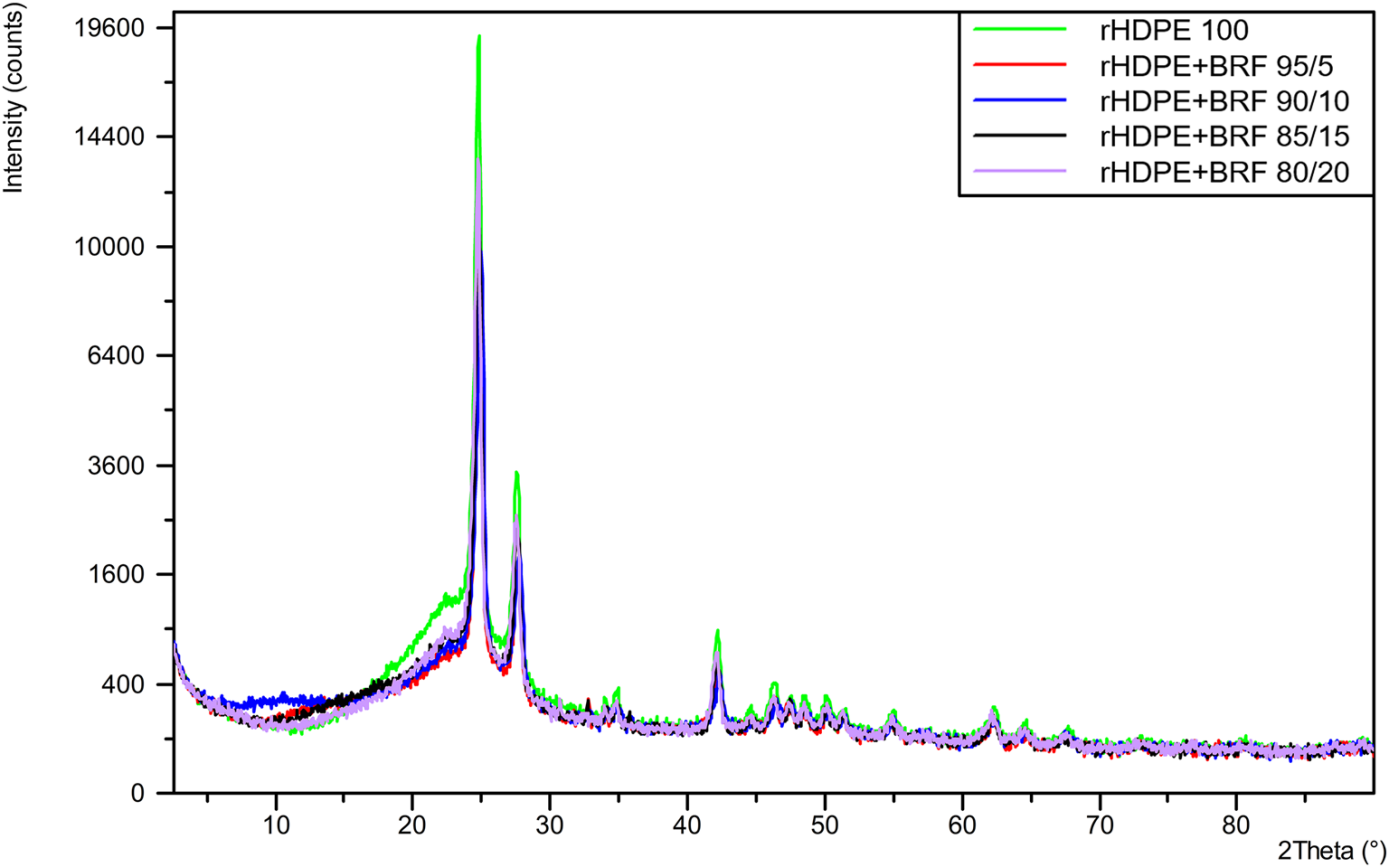
Comparison of all X-ray diffractograms obtained for rHDPE and rHDPE + BRF composites.

Figure 4 shows there are no relevant changes between the XRD results of the different formulations. The characteristics peaks of polyethylene can be seen in all XRD patterns over $2\theta$ at about 21.5°, 23,9°, and 36.2°, which correspond to the (110), (200), (020) lattice places, indicating that the HDPE structure corresponds to an orthorhombic structure^47^. Some other small peaks that appear in the range of 30°–50° indicate the semi-crystalline nature of rHDPE^38,48^.

All the graphs maintain a similar spectrum; however, it can be noted that the peak position of three rHDPE peaks in this study is slightly shifted to a higher angle, which can be assigned to the unit cell distortion of HDPE by impurities filler inclusions^47^. Besides that, it can be noted that there are no horizontal shifts of the characteristic peaks of the graphs of the compounds concerning the net rHDPE, i.e., there is no phase change in the crystalline structures present^49^. With the addition of BRF, the intensities of the crystal peaks decreased slightly, reflecting the filler's possible effective dispersion in the polymer matrix and indicating a mild reduction in the rHDPE crystallinity^38^.

Figure S3 in the Supplementary Information File shows some uncharacteristic peaks according to the literature. This is because it is a recycled polymer containing impurities reflected in the spectrum.

**Figure 5:**
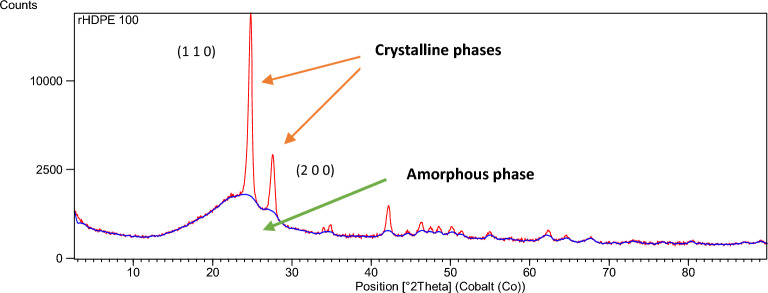
The diffractogram of rHDPE and its main characteristic peaks.

Figure 5 presents the X-ray diffractogram of rHDPE. This semi-crystalline material shows peaks that are characteristic of the internal structure. Wide and narrow peaks can be associated with amorphous components^50,51^.

**Figure 6:**
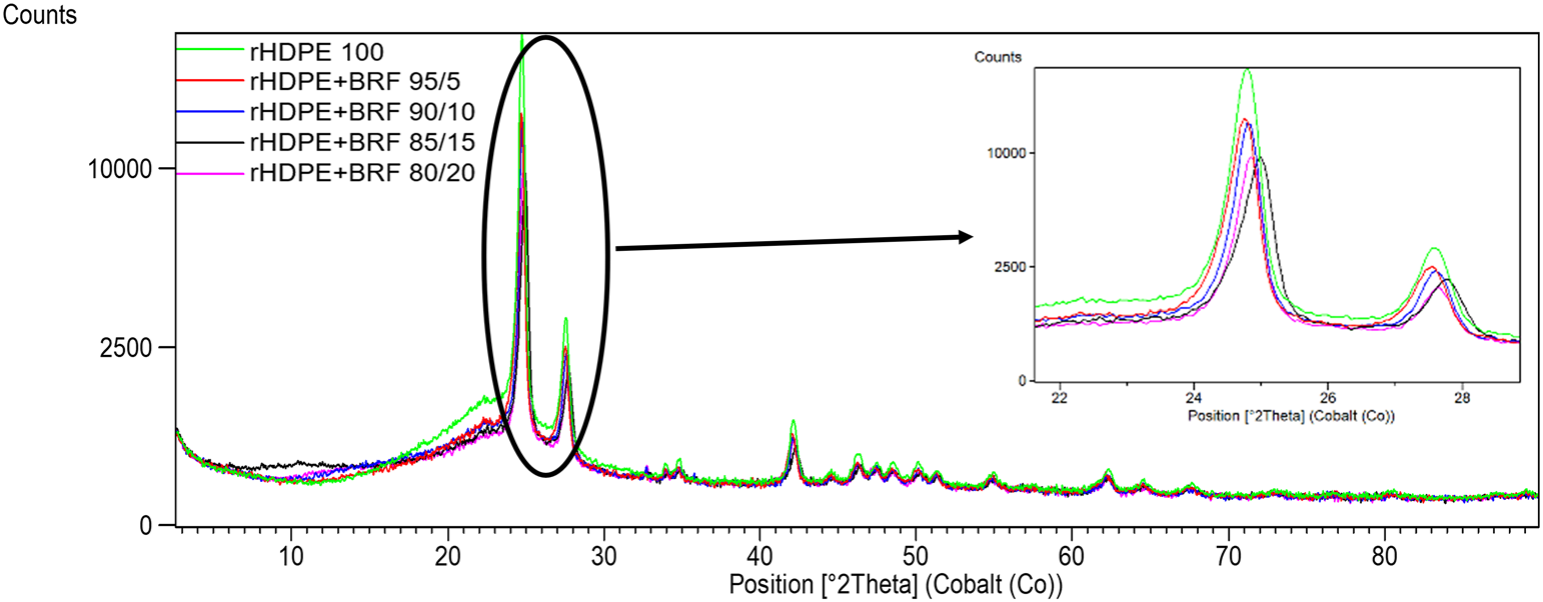
XRD of rHDPE and rHDPE + BRF composites.

The HDPE polymer presents two characteristic peaks of polyethylene (C_2_H_4_). These peaks can be found at 2θ of 24.78° and 27.58°^50–52^. Figure 2 shows the XRD results of rHDPE composites. The intensity of the peaks is reduced with the addition of the banana rachis. Each sample has a different percentage of crystallinity, as seen in Fig. 6.

Table 3 shows the crystalline planes of virgin polyethylene, considering the intensity of the different peaks and at different compositions.

**Table 3:** XRD analysis of the main crystalline groups of the polymeric matrix.

Sample	2θ		Intensity (counts)	
	Peak (I)	Peak (II)	Peak (I)	Peak (II)
rHDPE 100	24.784	27.578	18,298.8	2783.1
rHDPE + BRF 95/5	24.75	27.532	12,710.5	2074.2
rHDPE + BRF 90/10	24.809	27.602	12,338.8	1838.5
rHDPE + BRF ​85/15	24.98	27.758	9059.6	1548.4
rHDPE + BRF 80/20	24.847	27.632	9259.8	1257.5

The following equation has been used to determine the crystallinity percentage of the samples^51^:

3 $${\% }Cristallinity=\frac{Area\, under \,crystalline \,peaks}{Total \,area \,underall \,peaks}\times 100{\%}$$

Table 4 shows the data for the crystallinity of each composite sample. The percentage of the additive (in this case, BRF) directly affects the material's crystallinity (matrix)^51^. The loss in crystallinity concerning the BRF loading can be visually tracked, considering the decrease in peak intensities/counts as the BRF content increases in composite (see Fig. 6).

**Table 4:** The crystallinity value of each sample using XRD data.

HDPE	rHDPE 100	rHDPE + BRF 95/5	rHDPE + BRF 90/10	rHDPE + BRF ​85/15	rHDPE + BRF 80/20
Area (amorphous peaks)	11,780.8	9965.3	10,995.6	11,475.6	9981.7
Area (crystalline peaks (I))	9740.9	6475.7	5775.6	4819.6	4237.1
Area (crystalline peaks (II))	1701.2	1173	1065.8	960.3	678
Total area	23,222.9	17,614.0	17,837	17,255.5	14,896.8
Crystallinity (%)	49.27	43.42	38.36	33.50	32.99
Crystallinity reduction	0	11.87	22.14	32.01	33.04

### Thermogravimetric analysis (TGA)

**Figure 7:**
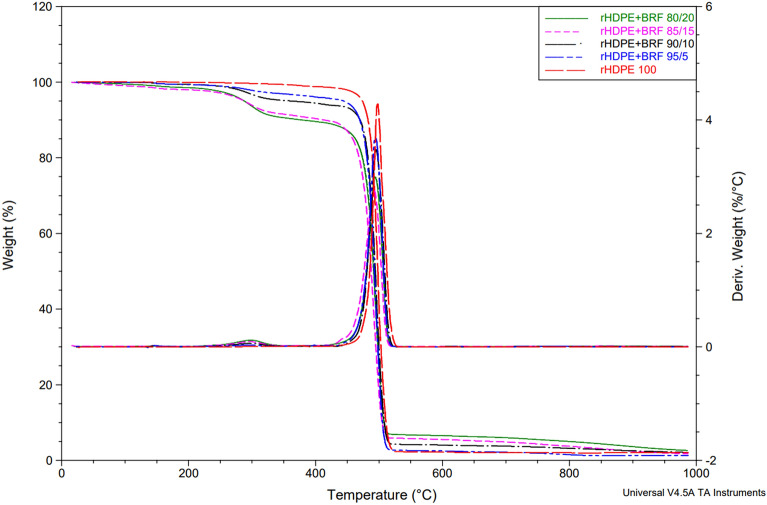
Comparison of all the TGA results obtained for rHDPE and rHDPE + BRF composites.

Figure 7 shows the comparison between all TGA curves of the different blends. There are two predominant thermal stages in fiber-containing composite systems. One degradation stage corresponds to the added fiber, and the other corresponds to the rHDPE polymeric matrix. In the rHDPE curve, the initial loss weight of 1.2% corresponds to moisture content. Also, the primary degradation stage presents a weight loss of 96% over 470–525  °C due to the rHDPE decomposition. These results are similar to those reported in the literature^19^.

Hemicellulose and cellulose are significant components in banana fiber, and their degradation temperature can be observed in Figure S4 (in the Supplementary Information File), which has a maximum of 292.14 °C, as reported in the literature^35^. Figure S4 in the Supplementary Information File shows BRF degradation, which weight loss initiated with dehydration of 10% in the temperature region 20–120 °C. The noticeable degradation occurs in the range of 200–400 °C due to cellulose and hemicellulose pyrolyzed. Finally, lignin degradation crops up over the temperature range of 100–900 °C^36^.

As the percentage of banana fiber in the polymeric matrix increases, the peak corresponding to the hemicellulose and cellulose degradation, which appears between 218 and 398 °C, increases. The proportional increase between this significant peak and the fiber percentage addition indicates a good dispersion of fiber added into the polymeric matrix.

Lignin is also a component of BRF and has a slow degradation, which occurs from 100 to 900 °C^35^. This is evident in the area where the curve decreases slowly from 500 to 900 °C in all cases. It is observed that the magnitude of the lignin degradation curve increases proportionally with the percentage of fiber, which is expected. The residue of each sample showed a proportional increase concerning the BRF content. In the rHDPE 100 curve, the residual percentage is 1.993% and increases to 2.625% with the addition of 20% fiber. The residue percentage of the banana rachis is 23.65% (see Figure S4 in the Supplementary Information File), so it is evident that this residue increments in the composites proportional to the filler increase.

### Rheology test

**Figure 8:**
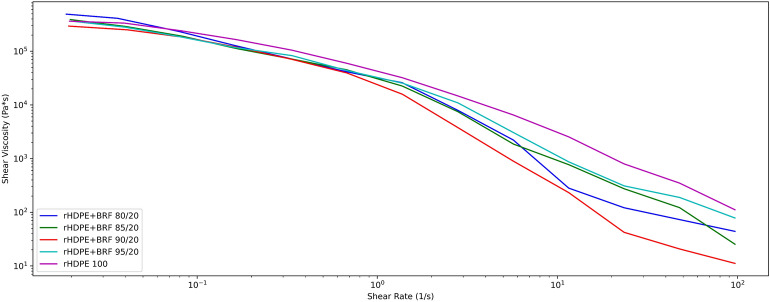
Comparison of all the rheology results obtained for rHDPE and rHDPE + BRF composites.

Figure 8 shows that the curve trend of each formulation presents a pseudoplastic behavior^53^. The addition of fiber in the polymer matrix decreases the viscosity of the composite as the shear rate increases. The composites only at low shear rate values present a slightly higher viscosity to neat rHDPE^54^. Neat rHDPE and rHDPE + BRF 95/5 composite present similar flow behavior in the analyzed shear rate range. Slightly higher viscosity values have been obtained starting from the rHDPE + BRF90/10 sample compared to neat rHDPE at low frequencies. It could be due to the fiber addition restricting the polymer chains, preventing the composite fluidity, and incrementing its viscosity^54^.

### Mechanical properties

Table 5 shows the mechanical properties (Tensile and impact results) of rHDPE composites at a variable weight percentage of BRF (5–20 wt%). Tensile strength and elongation at break decrease as the filler percentage increases. The rHDPE + BRF 80/20 sample showed σ_max_ and δ present a reduction of 20% and 97%, respectively, compared with the neat rHDPE. The decrease in tensile strength and elongation was also observed by Satapathy et al*.*^35^. The reduction might be caused due to the broken-down structural integrity by the incorporation of fibers into the matrix. Besides that, stress and repulsions might also be formed between disparate fiber and matrix, which results in weak mechanical properties. However, an increase in tensile modulus values was observed with increased BRF content. The Tensile Modulus increased by 14% with an increase in the BRF content to 20 wt% compared with the rHDPE matrix. A higher BRF content in the polymer matrix increases the composite stiffness. This result is consistent with the literature^2^. The increment in filler content acts as a defect in the microstructure because of the compatibility of the materials. The rHDPE/BRF composites demonstrated a significant reduction in the Izod Impact test when BRF content increased. The rHDPE + BRF 80/20 sample showed a decrease in impact resistance up to 50% compared with neat rHDPE. Similar results have been reported in the literature^35^ regarding the reduction of composites' energy absorption (toughness loss) by the presence of BRF particles. Some studies have shown that the chemical pre-treatment of the banana fiber or the use of coupling agents for different matrixes improves the fiber-matrix interfacial adhesion, increasing the composites' mechanical properties^5,20,35,55^.

**Table 5:** Mechanical properties of rHDPE/BRF composites (tensile strength (σ_max_), elongation at break (δ), tensile modulus (E), izod impact strength).

Sample	σ_max_ (MPa)	δ (%)	E (MPa)	Izod impact strength (J m^−2^)
rHDPE 100	24.10 ± 1.72	216.19 ± 51.2	1034.53 ± 33.11	125.1 ± 34.4
rHDPE + BRF 95/5	19.68 ± 1.12	36.67 ± 11.28	914.26 ± 49.27	65.1 ± 14.3
rHDPE + BRF 90/10	19.22 ± 1.95	20.53 ± 7.13	1023.44 ± 137.41	53.3 ± 1.1
rHDPE + BRF ​85/15	16.24 ± 0.42	21.84 ± 14.87	891.64 ± 24,58	42.5 ± 0.6
rHDPE + BRF 80/20	18.64 ± 1.1	6.65 ± 0.89	1182.37 ± 43.75	33.6 ± 4

### Scanning electron microscopy (SEM)

**Figure 9:**
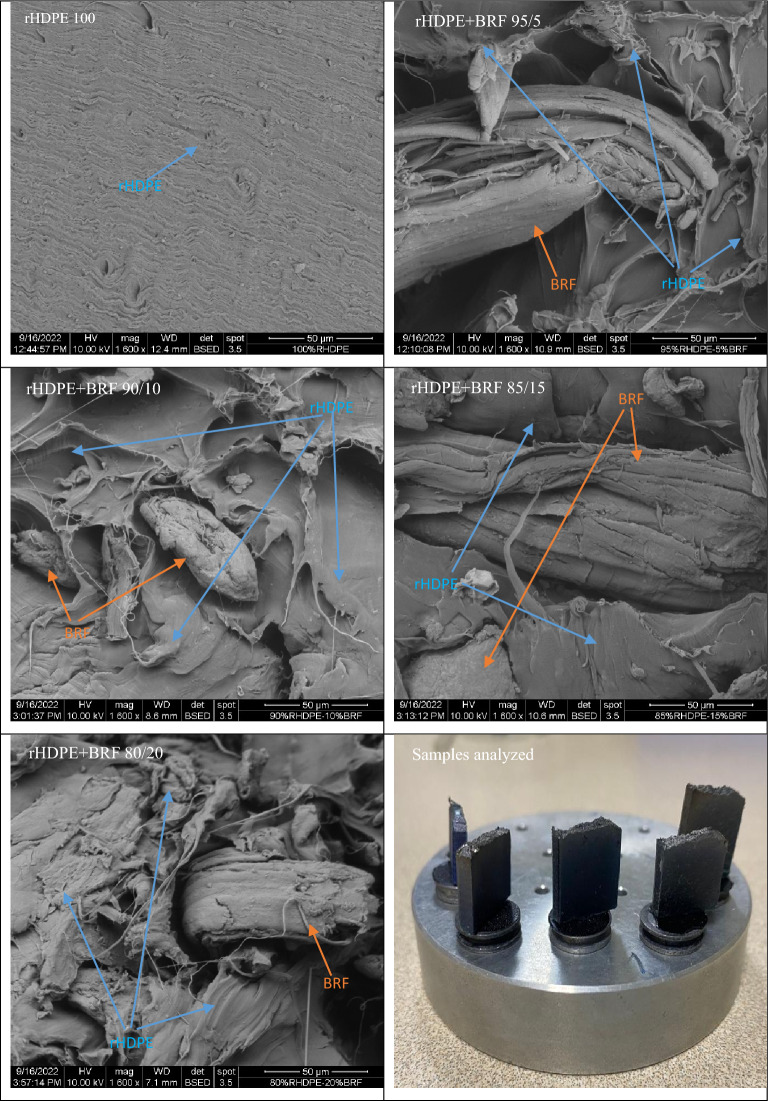
The microstructure of rHDPE and rHDPE + BRF composites and the shape of the samples' remaining parts analyzed after the IZOD impact test.

Figure 9 shows the image of edge-cut samples prepared without any treatment for SEM analysis and microstructures of pure rHDPE and its 5–20 wt% untreated banana rachis containing composites.

As seen in the figure, the rHDPE 100 sample shows a smooth surface when the sample is broken. On the other hand, SEM images of composites show a rough microstructure because of the voids formed in the microstructure due to the impregnation of BRF inside the rHDPE matrix. The microstructure of the points evidences the difference between the dimension and orientation of the fibers inside the matrix. It is clear that the fibers have caused stress between the polyethylene chains considering the average particle size of the BRF is 240 μm. Especially if the elongation at break values is revised, it is seen that the value decreases as BRF content increases. The low adhesion between the rHDPE and BRF phases is evident in the SEM images, and this phenomenon has been reported in the literature, supporting our findings^56^. Hence the flexibility of the rHDPE + BRF 80/20 decreases very drastically compared to the rHDPE 100 sample.

Widiastuti et al*.*^56^ clarified the decrease in mechanical properties of the bamboo fiber-rHDPE composites indicating the least interaction between the matrix and the fiber using SEM images. The micrographs given in that study are unclear due to low resolution, so it is very hard to reach the conclusions that the authors have reached based on SEM images. Nevertheless, in the current study, SEM images are self-explanatory, showing the low affinity between rHDPE and BRF, corroborating that the decrease in mechanical properties of the composites is proportional to the BRF content. Likewise, as seen in the images, some BFRs have very different shapes and orientations, forming kind of voids (see the micrograph of the rHDPE + BRF ​85/15 sample in Fig. 9) in the rHDPE matrix. This is another reason that rHDPE-BRF composites have lesser mechanical properties comparing pure rHDPE. Additionally, as the fibers have different shapes and sizes, their distribution in rHDPE is another issue that affects the properties of the composites.

### Life cycle assessment

**Figure 10:**
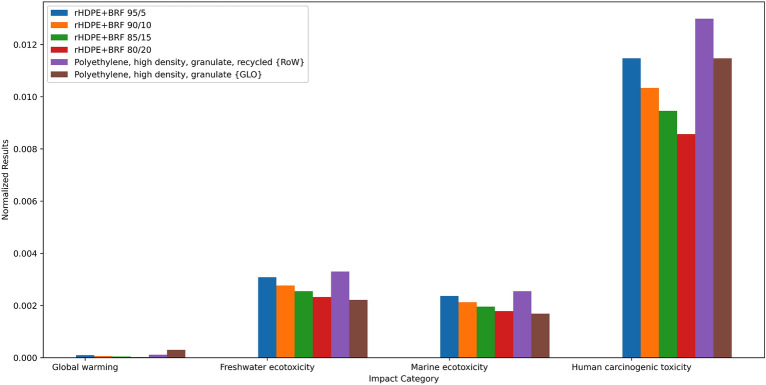
Normalized impact results for all rHDPE + BRF composites.

The life cycle assessment for the rHDPE composite showed the environmental impact of including the natural fibers from the banana rachis as reinforcement in the polymeric material. It showed an inverse proportionality between adding fiber and the environmental impacts. For instance, rHDPE + BRF 95/5 sample has 69% fewer carbon emissions than virgin polymer and 18% less impact than recycled polyethylene. Figure 10 shows the normalized environmental impacts for virgin, recycled, and HDPE composites. It can be noticed that the impact from virgin HDPE is higher than the composites. Recycled HDPE has a higher impact score in the ecotoxicity and human toxicity categories related to energy usage in the recycling process. The yellow bar represents the rHDPE + BRF 80/20 sample, which scored lowest in every category.

**Figure 11:**
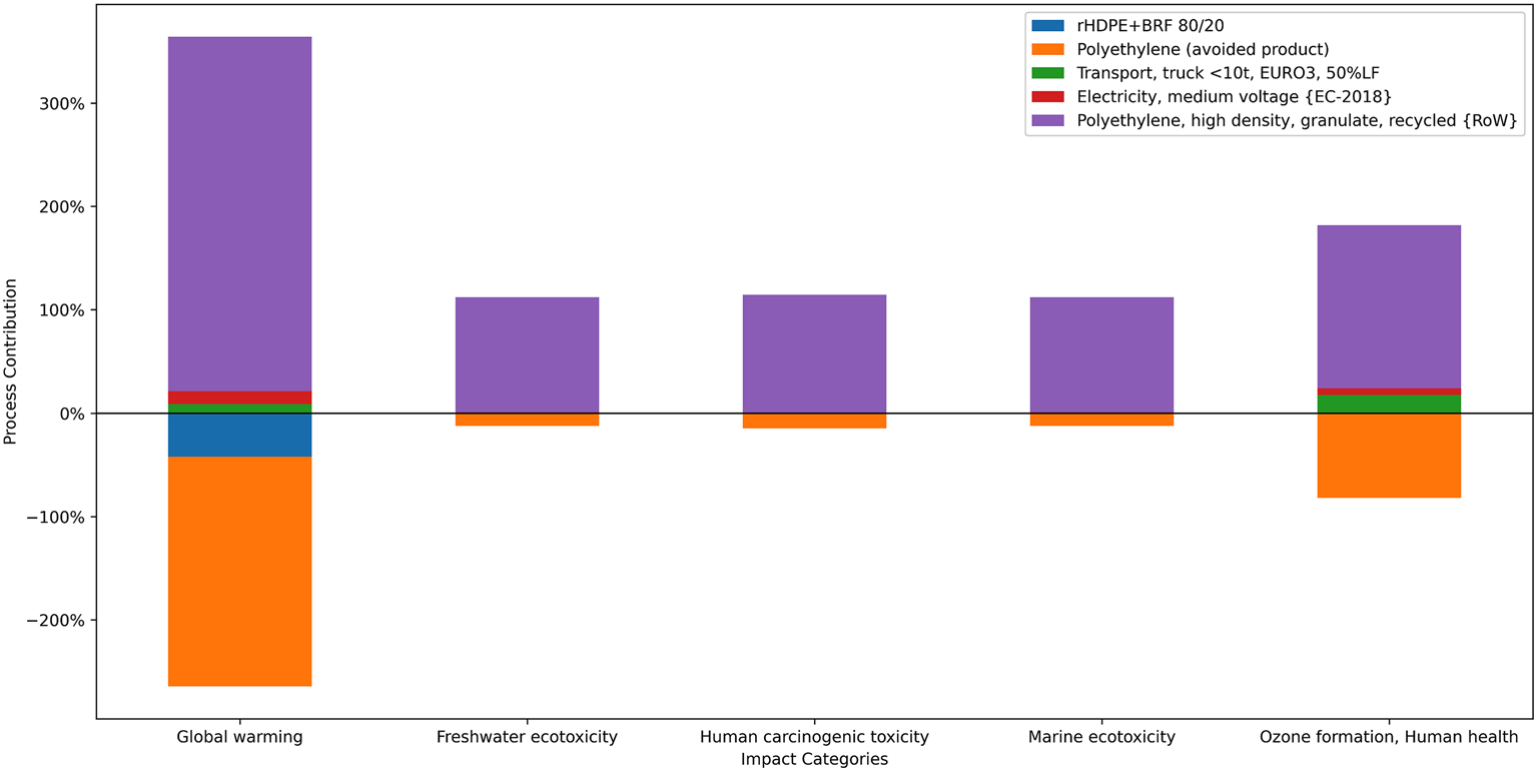
Process contribution of some impact categories for rHDPE + BRF 80/20 sample preparation.

Bataineh^57^ analyzed the recycling of HDPE and PET under the LCA methodology. The water usage for that study was 50% lower than the water used for the rHDPE-Banana Rachis 20%. This may come from the added electricity used for the composite elaboration. In addition, Bataineh^57^ estimated a carbon emission of 0.63 kg CO_2_ per kg of HDPE. This is 3.3 times more impact on this indicator. Figure 11 shows that recycled polyethylene is the most significant contributor in every impact category. The impact results found in the current study are less for each category because of the inclusion of banana rachis, which replaces some of the rHDPE.

Another study^58^ analyzed recycled HDPE for use as a net for agriculture with an emission of 0.553 kg CO_2_ per 130 g of rHDPE. This is 22.4 times more contribution to global warming than the composite that uses 20% of the banana rachis. In this sense, Malviya et al*.*^59^ agree that using natural fibers lowers the impact of Green House gases to the impact category. Another research^60^ compared the impact of using different polymers like HDPE and PLA and using banana fibers in the composite. The trend seems to agree with the literature; adding natural fibers does not affect or positively affect lowering CO_2_ emissions.

In the same way, a wood-polymer composite showed a reduction in the impacts when the proportion of wood doubled^61^. For instance, the increase of wood particles from 30 to 60% affected a 26% reduction in the global warming potential. Research shows that shifting from virgin HDPE to recycle HDPE has a positive impact^62^; consequently, further research can analyze the use of bio-based HDPE^63^ with the banana rachis for much better environmental performance.

## Conclusions

The composite materials based on different amounts of untreated BRFs and rHDPE have been successfully prepared and characterized by several methods, as mentioned earlier. The physical, mechanical, thermal, and rheological properties of BRF-based rHDPE composites were determined to evaluate the fiber's effect on the composite materials' final properties. Additionally, the environmental impact of the production of these BRF-based rHDPE composites has been investigated using a life cycle assessment approach.

Different BRF loadings in composites decrease the thermal stability, specifically, the initial/onset degradation temperature.

The crystallinity of the composites diminishes concerning the rise of the BRF content in the rHDPE matrix.

Almost all the mechanical properties of composites significantly diminish by incorporating different BRF loadings in the rHDPE matrix, while only the stiffness/tensile modulus enhances slightly.

Adding BRF into the polymeric matrix reduces the overall environmental impacts of the composites. Especially higher percentages of BRF in composites significantly reduce the amount of rHDPE necessary for the composite preparation, which is the main contributor in every impact category.

Further studies and considerations include chemical treatments of the BRF before its use, using compatibilizers to improve the fiber-matrix interfacial adherence, and preparation and use of nano BRF particles can be taken into account to improve the composites' mechanical properties.

### Supplementary Information

Supplementary Information.

## Data Availability

Additional data has been provided in the electronic supplementary material available for download.
