# Supplementary material for: Preparation, characterization, and life cycle assessment of banana rachis-recycled high-density polyethylene composites
Source: Sci Rep. 2023 Oct 2;13:16534. doi: 10.1038/s41598-023-42613-0 (PMC10545752; doi:10.1038/s41598-023-42613-0)
Supplement: Supplementary file 1 — Supplementary Information. [file 41598_2023_42613_MOESM1_ESM.docx]

**Preparation, characterization, and life cycle assessment of banana rachis-recycled high-density polyethylene composites**

Demis Cabrera^1,2^, Haci Baykara^1,3^[[1]](#footnote-1)^*^, Ariel Riofrío^3^, Mauricio Cornejo^1,3^, Julio Cáceres^3^

^1^Faculty of Mechanical Engineering and Production Science (FIMCP), ESPOL Polytechnic University, Guayaquil P.O. Box 09-01-5863, Ecuador

^2^Plastics Processing Laboratory (PPL), ESPOL Polytechnic University, Guayaquil, P.O. Box 09-01-5863, Ecuador

^3^CIDNA, ESPOL Polytechnic University, Guayaquil, P.O. Box 09-01-5863, Ecuador


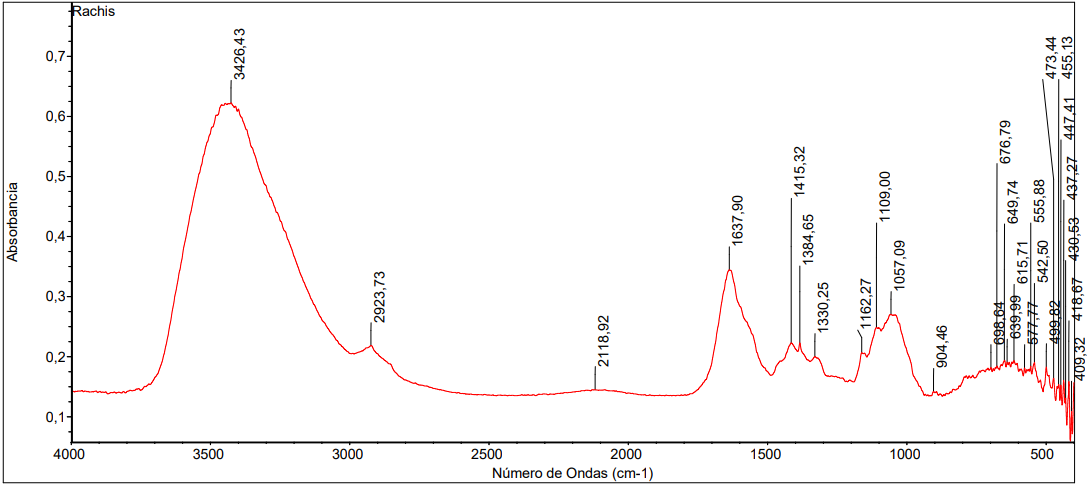


**Figure S1.** FTIR test results for banana rachis fiber


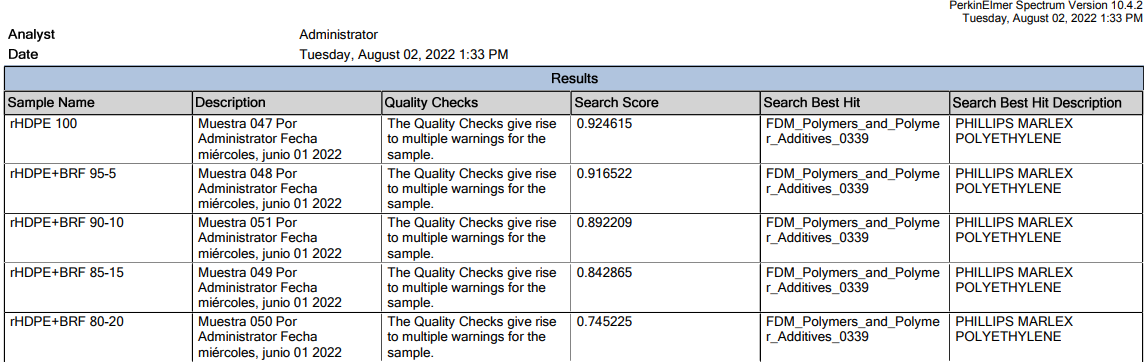


**Figure S2.** Similarity percentage of different composites compared with the pure HDPE using data of the PerkinElmer Spectrum Version 10.4.2 equipment.


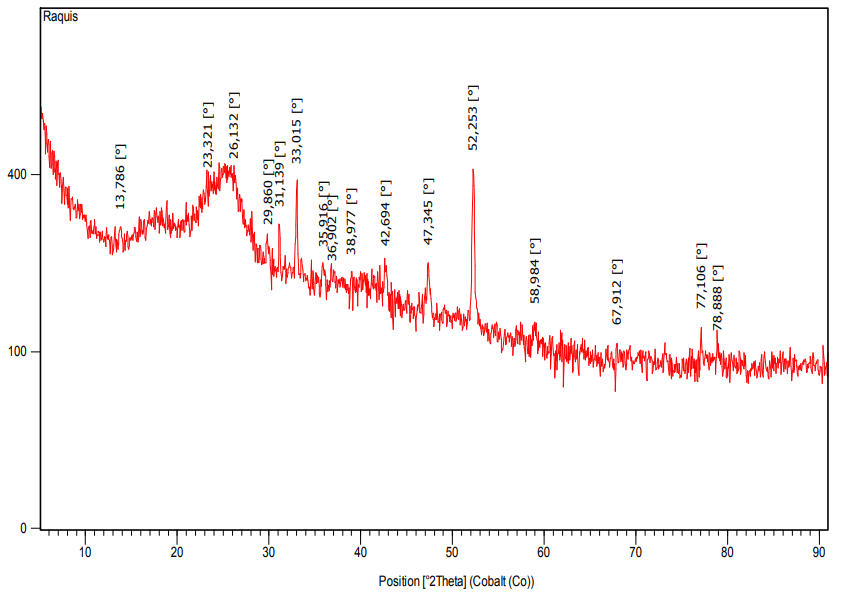


**Figure S3.** XRD test results for banana’s rachis fiber


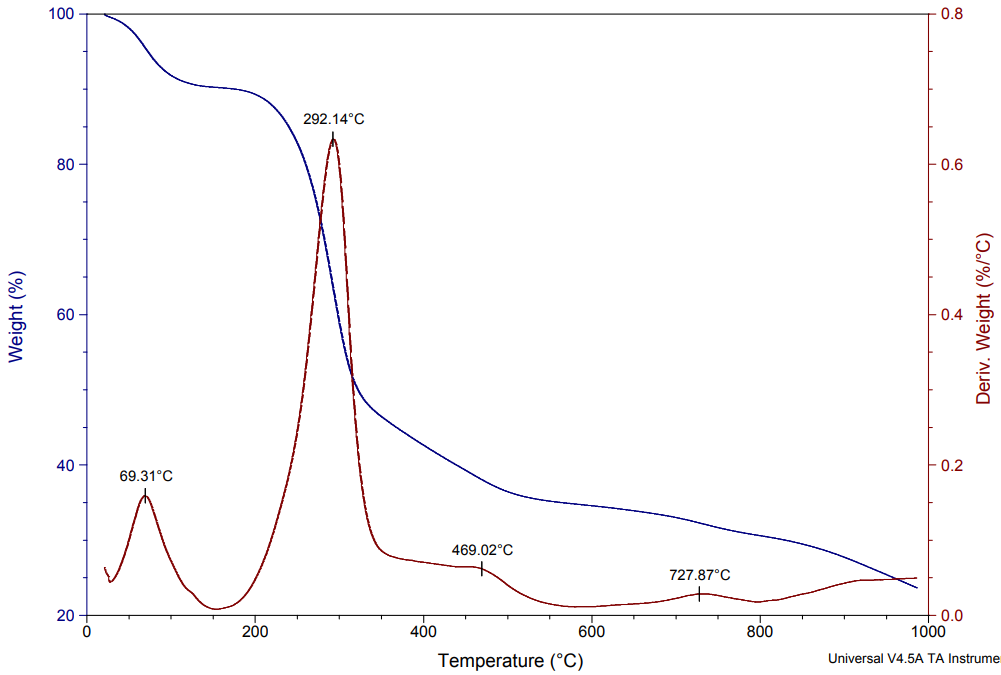


**Figure S4.** TGA results for banana’s rachis fiber.

1. * Corresponding author

   Email address: [hbaykara@espol.edu.ec](mailto:hbaykara@espol.edu.ec) (H.Baykara) [↑](#footnote-ref-1)
